# Does the TNM classification of solitary internal mammary lymph node metastases in breast cancer still apply?

**DOI:** 10.1007/s10549-016-4071-x

**Published:** 2016-12-03

**Authors:** V. Habraken, T. J. A. van Nijnatten, L. de Munck, M. Moossdorff, E. M. Heuts, M. B. I. Lobbes, M. L. Smidt

**Affiliations:** 1 Department of Surgery, Maastricht University Medical Center +, Maastricht, The Netherlands; 2 Department of Radiology and Nuclear Medicine, Maastricht University Medical Center +, P.O. Box 5800, 6202 AZ Maastricht, The Netherlands; 3 GROW – School for Oncology and Developmental Biology, Maastricht University Medical Center +, Maastricht, The Netherlands; 4 Department of Research, Netherlands Comprehensive Cancer Organisation, Utrecht, The Netherlands

**Keywords:** Breast cancer, Internal mammary lymph node, Neoplasm staging, Prognosis

## Abstract

**Purpose:**

TNM classification of solitary internal mammary lymph node metastases (IMLNMs) in breast cancer varies depending on their method of detection: sentinel lymph node biopsy (pN1b) or clinical examination including radiological and/or physical examination (pN2b). This study aimed to evaluate whether there is a difference in prognosis between both groups.

**Methods:**

Data of all patients diagnosed with primary invasive epithelial breast cancer between 2005 and 2008 were obtained from the Netherlands Cancer Registry. Patients with IMLNMs were divided in groups according to their pN1b and pN2b status. The main outcome measures disease-free survival (DFS) after 5 years and overall survival (OS) after 8 years were analyzed using Kaplan–Meier survival analysis. Cox regression analysis was used to determine independent predictors for DFS and OS.

**Results:**

A total of 73 patients with pN1b status and 28 patients with pN2b status were included. DFS rate was 74.1% in the pN1b group compared to 85.0% in the pN2b group (*p* = 0.211). Regarding OS, 20.5% (pN1b) and 25.0% (pN2b) of the patients deceased within 8 years of follow-up (*p* = 0.589). In multivariable cox regression analysis, nodal status was not statistically significant for DFS (HR 0.29 [95% CI 0.04–2.33], *p* = 0.244) or OS (HR 1.04 [95% CI 0.37–2.89], *p* = 0.947).

**Conclusions:**

Although the TNM classification considers pN1b and pN2b to be distinct prognostic entities, we did not observe any prognostic differences between these groups. Therefore, solitary IMLNMs may be regarded as a single category in the future and revision of TNM classification should be considered.

## Introduction

In breast cancer staging, TNM classification is used to determine the anatomic extent of the disease and consequently identify specific subgroups with different prognoses [1, 2]. Pathologic nodal staging is an important element in this classification as the presence of regional nodal metastases is associated with impaired survival [3]. These metastases can occur not only in axillary but also in extra-axillary lymph nodes, such as intramammary, periclavicular, interpectoral, and internal mammary lymph nodes.

Pathological nodal staging of internal mammary lymph node metastases (IMLNMs) has changed over time. In the fourth (1987) and fifth edition (1997) edition of TNM classification, all IMLNMs were classified as pN3, because by that time IMLNMs were considered of great importance in formulating the prognosis of patients [4, 5]. Since the introduction of the sixth edition in 2002, IMLNMs are divided into pN1b, pN1c, pN2b, or pN3b status depending on their method of detection and possible concurrent axillary lymph node metastases [6, 7]. IMLNMs may be detected by physical and/or radiological examination or by sentinel lymph node biopsy (SLNB) [8]. Nowadays, solitary IMLNMs, in the absence of axillary lymph node metastases, are considered pN1b when detected at SLNB and pN2b when detected at clinical examination (including physical and/or radiological examination) [7, 9, 10].

Dividing solitary IMLNMs based on the method of detection, TNM implies a difference in prognosis between both groups. Therefore, the aim of this study was to evaluate whether a true difference in prognosis exists between pN1b and pN2b status.

## Methods

### Data collection

Data of all patients diagnosed between 2005 and 2008 with primary invasive epithelial breast cancer were obtained from the Netherlands Cancer Registry (NCR), which is managed by the Netherlands Comprehensive Cancer Organisation (IKNL). The NCR ensures a high-quality data collection using specially trained employees who extract patient, tumor, and treatment characteristics directly from the patient records. Groups were defined according to pN1b (IMLNMs detected at SLNB) and pN2b (IMLNMs detected at clinical examination) nodal status. Characteristics collected were age, tumor characteristics (size, location, stage, grade, subtype, and receptor status), and treatment characteristics (adjuvant chemotherapy, targeted therapy, endocrine therapy, and radiation therapy).

### Treatment

During the study period, the Dutch national guideline of 2005 was in use [11]. This guideline recommended regional treatment depending on nodal status: SLNB was indicated in clinically node-negative patients, based on physical examination, with axillary ultrasound being commonly used but not mandatory at that time. Clinically node-positive (N+) patients, patients with positive SLNB, or patients with a contraindication for SLNB underwent axillary lymph node dissection (ALND).

In all patients who underwent lumpectomy, whole-breast irradiation was indicated. After mastectomy, chest wall irradiation was indicated in the case of irradical resection, pT4 tumors, and involvement of the pectoral muscle or skin. For pT3 tumors, chest wall irradiation was considered individually. Irradiation of regional nodal fields was included in case of four or more axillary lymph node metastases or involvement of top axillary lymph nodes after ALND. The recommended dose was 45–50 Gy in 5 weeks, with a boost to 60–70 Gy when residual tumor was present.

Chemotherapy was recommended in all premenopausal N+ women and in postmenopausal N+ women aged 50–69 with estrogen receptor (ER)- and progesterone receptor (PR)-negative tumors. Furthermore, chemotherapy was considered in physically fit postmenopausal N+ women aged 50–59 with ER- and PR-positive tumors and in N+ women aged 60–69 if four or more regional lymph nodes were involved. Chemotherapy regimen consisted of five courses of 5-Fluorouracil, Epirubicin, Cyclophosphamide (FEC) or six courses of Taxotere, Adriamycin, and Cyclophosphamide (TAC). Targeted therapy (trastuzumab) was recommended in selected cases in addition to chemotherapy in case of human epidermal growth factor 2 receptor amplification (HER2+). Endocrine therapy was recommended for all ER- and/or PR-positive tumors.

### Statistical analysis

All analyses were performed using IBM SPSS Statistics version 23.0 (IBM Corporation, Armonk, New York, USA) and *p* values <0.05 were considered statistically significant. Differences between pN1b and pN2b groups with regard to patient, tumor, and treatment characteristics were tested using the Fisher’s Exact Test and Pearson Chi-square test for categorical variables and Mann–Whitney *U* test for continuous variables.

The main outcome measures were disease-free survival (DFS) after 5 years and overall survival (OS) after 8 years. DFS was defined as the absence of any first local, regional, or contralateral recurrence, distant metastasis, or mortality within 5 years. DFS rate included all patients without any event, who visited the hospital in the fifth year after diagnosis for regular check-up. OS was defined as the time interval between date of diagnosis and date of death or date of emigration, as obtained from the Municipal Personal Records Database and completed until December 31, 2014. Patients were censored at the date of their first event, date of last follow-up, date of death, or date of emigration, whatever came first. Patients without follow-up data were excluded from DFS analysis. DFS and OS were analyzed using Kaplan–Meier survival analysis and compared with the log-rank test.

Univariable and multivariable Cox regression analyses were used to determine relevant predictors for DFS and OS. Outcome measure was hazard ratio (HR) with corresponding 95% confidence intervals (CI). Due to the limited number of events, multivariable cox regression could only be performed with a limited number of variables [12]. Nodal status together with the most significant variables in univariable cox regression was selected for multivariable cox regression.

## Results

### General characteristics

**Fig. 1:**
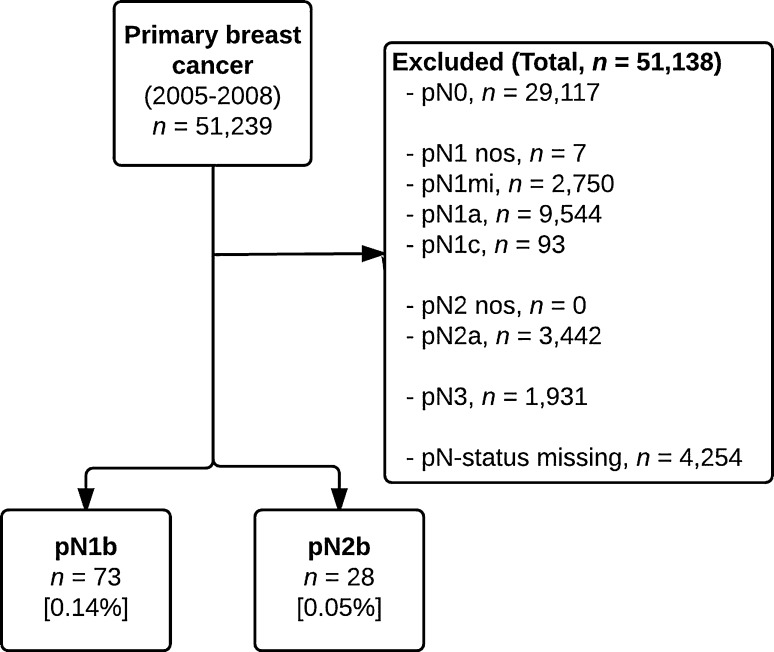
Flowchart of patient selection. *nos* indicates not otherwise specified, *mi* indicates micrometastases, *pN3* includes pN3a, pN3b, and pN3c

Between 2005 and 2008, a total of 51,239 patients were diagnosed with primary invasive epithelial breast cancer. After selection for pN1b (*n* = 73, 72.3%) and pN2b (*n* = 28, 27.7%) status, a total of 101 patients remained, comprising 0.2% of the total population (Fig. 1). In comparison to pN1b status, pN2b was associated with lower rates of pT0-1 stage carcinoma (32 vs 59%, *p* = 0.016), lower rates of grade 1–2 carcinoma (32 vs 63%, *p* = 0.005), and larger mean tumor size (28 vs 20 mm, *p* = 0.008). A detailed overview of baseline patient, tumor, and treatment characteristics is shown in Table 1.

**Table 1:** Patient demographics and characteristics of tumor and treatment subdivided according to pN1b and pN2b status

	pN1b (*n* = 73)	pN2b (*n* = 28)	*p* value
Mean age, years (SD)	55 (14)	58 (17)	0.693
Mean tumor size, mm (SD)	20 (11)	28 (15)	0.008
pT-stage, *n* (%)			
T0–1	43 (59)	9 (39)	0.016
T2–4	30 (41)	14 (61)	0.419
Unknown	–	5	–
Tumor type, *n* (%)			
Ductal	54 (74)	19 (68)	0.539
Lobular	7 (10)	4 (14)	0.492
Mixed ductal & lobular	4 (5)	3 (11)	0.393
Other	8 (11)	2 (7)	0.722
Grade, *n* (%)			
1–2	46 (67)	9 (38)	0.005
3	23 (33)	15 (62)	0.040
Unknown	4	4	–
Receptor status, *n* (%)			
ER+, PR+, HER2−	39 (56)	16 (63)	0.737
ER+, PR−, HER2−	8 (11)	3 (11)	1.000
ER+, HER2+	9 (13)	3 (11)	1.000
ER−, HER2+	3 (4)	3 (11)	0.344
Triple negative	11 (16)	1 (4)	0.171
Unknown	3	2	–
Chemotherapy, *n* (%)	44 (60)	17 (61)	0.968
Radiation therapy, *n* (%)	55 (75)	19 (68)	0.447
Trastuzumab, *n* (%)	13 (18)	3 (11)	0.546
Endocrine therapy, *n* (%)	51 (70)	19 (68)	0.845

*SD* standard deviation, *pT*-*stage* pathologic tumor stage, *ER* estrogen receptor, *PR* progesterone receptor, *HER2* human epidermal growth factor receptor 2

### Disease-free survival (DFS)

**Fig. 2:**
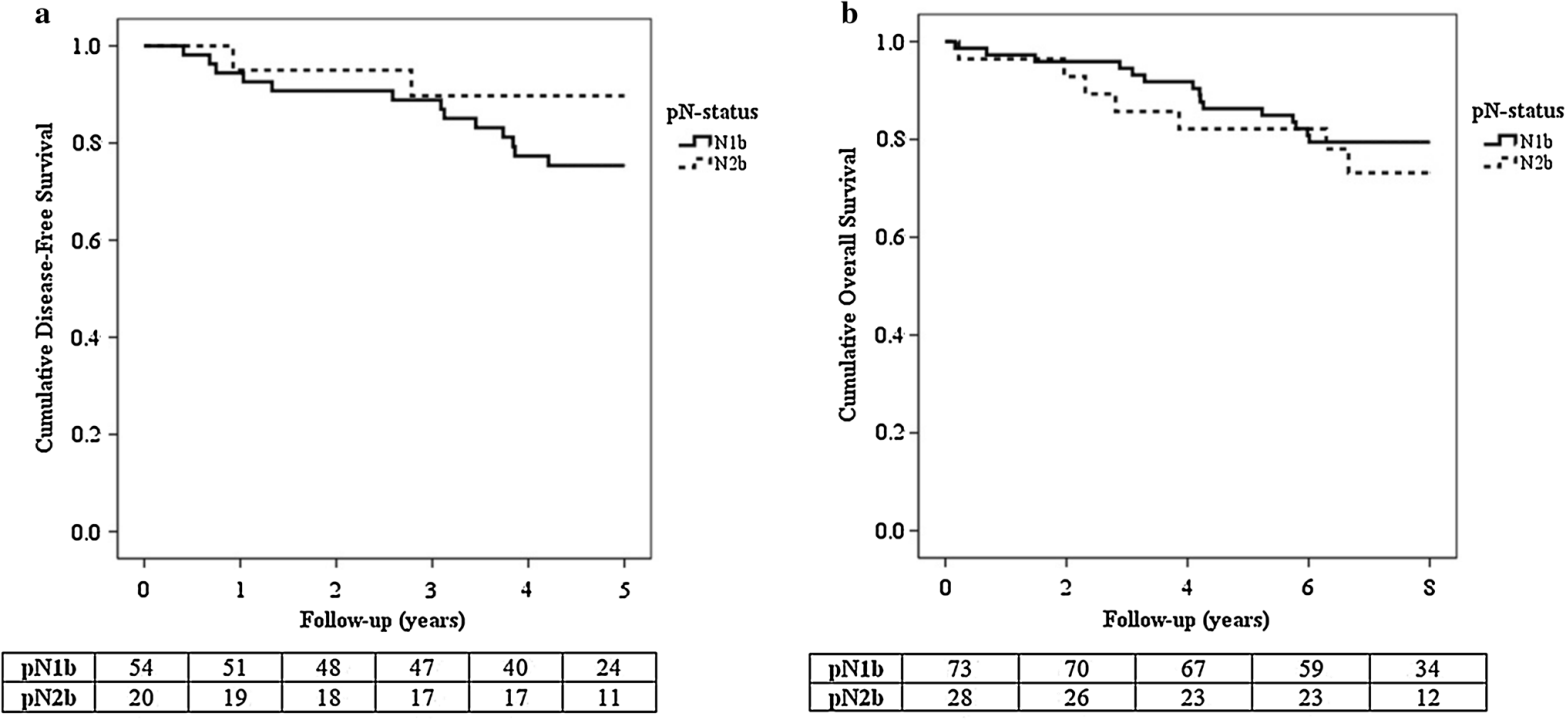
Kaplan–Meier survival curves of disease-free survival (**a**) and overall survival (**b**)

Five-year follow-up data were complete for 54 patients (74.0%) in the pN1b group and 20 patients (71.4%) in the pN2b group. An event occurred in 13 patients (24.1%) in the pN1b group compared to two patients (10.0%) in the pN2b group (*p* = 0.211) (Fig. 2a). DFS rate was 74.1% in the pN1b group and 85.0% in the pN2b group.

When taking the effect of endocrine therapy and triple-negative subtype into account in multivariable Cox regression analysis, pN2b status was not significantly different compared to pN1b status (HR 0.29 [95% CI 0.04–2.33], *p* = 0.244). Neither endocrine therapy nor triple-negative subtype was identified as an independent predictor for improved DFS (HR 0.46 [95% CI 0.12–1.86], *p* = 0.277 and HR 1.56 [95% CI 0.35–7.06], *p* = 0.561, respectively) (Table 2).

**Table 2:** Univariable and multivariable Cox regression analysis for disease-free survival

	Univariable Cox regression		Multivariable Cox regression	
	HR [95% CI]	*p* value	HR [95% CI]	*p* value
pN1b	Reference		Reference	
pN2b	0.40 [0.09–1.77]	0.227	0.29 [0.04–2.33]	0.244
Tumor size (per mm increment)	1.04 [1.00–1.07]	0.051		
pT-stage				
T2–4 versus T0–1	1.96 [0.71–5.42]	0.194		
Tumor grade				
3 versus 1–2	1.07 [0.37–3.09]	0.897		
Triple-negative subtype				
Yes versus no	3.58 [1.10–11.63]	0.034	1.56 [0.35–7.06]	0.561
Radiation therapy				
Yes versus no	1.13 [0.36–3.54]	0.838		
Chemotherapy				
Yes versus no	1.21 [0.44–3.35]	0.709		
Endocrine therapy				
Yes versus no	0.25 [0.09–0.70]	0.008	0.46 [0.12–1.86]	0.277
Trastuzumab				
Yes versus No	0.33 [0.04–2.47]	0.200		

*HR* hazard ratio, *CI* confidence interval, *pT*-*stage* pathological tumor stage

### Overall survival (OS)

Median follow-up time of patients was 7.7 years (range 59 days–9.9 years). After 8 years of follow-up, 15 patients (20.5%) in the pN1b group and seven patients (25.0%) in the pN2b group were deceased (*p* = 0.589) (Fig. 2b).

When taking the effect of tumor size (per mm increment), endocrine therapy, and trastuzumab into account in multivariable Cox regression analysis, pN2b status still was not significantly different compared to pN1b status (HR 1.04 [95% CI 0.37–2.89], *p* = 0.947). Tumor size (HR 1.02 [95% CI 1.00–1.05], *p* = 0.117), endocrine therapy (HR 0.40 [95% CI 0.15–1.04], *p* = 0.060), and trastuzumab (HR 0.26 [95% CI 0.04–1.98], *p* = 0.192) did not have a statistically significant influence on OS (Table 3).

**Table 3:** Univariable and multivariable Cox regression analysis for overall survival

	Univariable Cox regression		Multivariable Cox regression	
	HR [95% CI]	*p* value	HR [95% CI]	*p* value
pN1b	Reference	0.590	Reference	0.947
pN2b	1.28 [0.52–3.14]		1.04 [0.37–2.89]	
Tumor size (per mm increment)	1.04 [1.01–1.06]	0.003	1.02 [1.00–1.05]	0.117
pT-stage^a^				
T2–4 versus T0–1	2.19 [0.91–5.29]	0.082		
Tumor grade				
3 versus 1–2	1.73 [0.67–4.49]	0.259		
Triple-negative subtype				
Yes versus no	2.22 [0.74–6.71]	0.156		
Radiation therapy				
Yes versus no	0.85 [0.31–2.30]	0.748		
Chemotherapy				
Yes versus no	1.06 [0.45–2.48]	0.897		
Endocrine therapy				
Yes versus no	0.30 [0.13–0.69]	0.005	0.40 [0.15–1.04]	0.060
Trastuzumab				
Yes versus no	0.22 [0.03–1.63]	0.138	0.26 [0.04–1.98]	0.192

*HR* hazard ratio, *CI* confidence interval, *pT*-*stage* pathological tumor stage

^a^Excluded from multivariable analysis due to collinearity with tumor size

## Discussion

According to the current TNM classification, patients with solitary IMLNMs are considered pN1b when detected during SLNB and pN2b when observed during clinical examination (including radiologic and/or physical examination), suggesting a prognostic difference between these two groups [1, 2, 6]. However, our study demonstrated that both DFS after 5 years (*p* = 0.211) and OS after 8 years (*p* = 0.589) were not significantly different between both groups. Consequently, it is questionable whether the current TNM classification of IMLNMs is still appropriate.

The comparable prognosis of the pN1b and pN2b group in our study can be explained by the great improvements in imaging modalities over the last decade. In the past, clinical detection of IMLNMs was mostly restricted to large internal mammary lymph nodes found during physical examination (and later additional ultrasound if indicated). Consequently, IMLNMs detected during physical examination were much larger and thus associated with worse prognosis than IMLNMs detected during SLNB. In distant past, 10-year overall survival ranged from 0 to 61% in patients with IMLNMs compared to our cohort of patients with SLNB-detected IMLNMs, of which only 20.5% of the patients deceased after 8 years of follow-up [6, 13, 14]. Possible explanations for improved overall survival can be the introduction of other systemic regimen, such as trastuzumab, or detecting smaller IMLNMs with SLNB. Nowadays, the size of internal mammary lymph nodes detected using state-of-the-art imaging techniques such as PET/CT and MRI approaches the size of internal mammary nodes visualized during SLNB [15–17]. This suggests comparable prognosis of SLNB-detected IMLNMs and imaging-detected IMLNMs.

Routine evaluation of IMLNMs is controversial and is currently not recommended. The overriding arguments against routine evaluation of IMLNMs include their low incidence, their very limited impact on prognosis and treatment strategy, and the fact that tissue sampling is rather complex and associated with a risk of morbidity [13, 18, 19]. However, detection of IMLNMs during radiological examinations and SLNB will continue to occur and possibly even increase with improving accuracy of these techniques. Their unambiguous and accurate classification will remain important as the detection of IMLNMs may alter nonsurgical treatment in patients [20]. Current guidelines advise internal mammary irradiation in all patients with histologically proven and/or PET-positive IMLNMs and in patients with N2 status additional radiotherapy of the periclavicular region and/or thoracic wall can be advised [9, 10, 21]. A previous study by Heuts et al. demonstrated that adjuvant treatment plans were changed in only 3.4% (27/789) of the patients based on the presence of IMLNMs [20]. If TNM classification would be adapted by including all isolated IMLNMs in one group, then additional radiation therapy, besides internal mammary irradiation, could be omitted in these patients.

A major strength of this retrospective study is the use of a large population-based dataset from the Netherlands Cancer Registry providing patient, tumor, and treatment characteristics. However, as metastatic spread to the internal mammary lymph node chain is rare, a limited number of patients were only available per subgroup [22]. Early surgical series showed internal mammary involvement in 9.1% of patients undergoing extended radical mastectomy [23]. According to our study, solitary IMLNMs were reported in only 0.2% of the population suggesting that IMLNMs may currently be underdiagnosed. Firstly, routine evaluation of IMLNMs is not recommended. Secondly, according to literature, superficial tracer injection (intradermal or periareaolar) often used during SLNB yields a lower visualization rate of internal mammary sentinel lymph nodes compared to intraparenchymal tracer injection (peritumoral, intratumoral, or subtumoral) [24]. All in all, the results of this study should be interpreted in the context of this small study population.

Furthermore, the staging technique used to classify patients as pN2b in our cohort was unknown. As a consequence, there was no distinction in our cohort of pN2b patients detected by for instance physical examination, ultrasound, MRI, or PET-CT. Yet, a previous study of Jochelson et al. demonstrated a difference in the prevalence between several imaging techniques for detecting internal mammary adenopathy [17].

Another study limitation may be the completeness of data. Nodal status was missing in over 4000 patients (10.3%) of the overall population of patients diagnosed with breast cancer in the Netherlands between 2005 and 2008. However, subclassification of pN1 (into pNmi, pN1a, pN1b, and pN1c) and pN2 (into pN2a and pN2b) status seems to be accurately registered as in the pN1 group only seven patients were classified as pN1 not otherwise specified and none in the pN2 group (Fig. 1). Therefore, registration of nodal status in our cohort was performed adequately.

In conclusion, our study did not observe any difference in prognosis between pN1b and pN2b in terms of DFS and OS. Since coincidental detection of IMLNMs during SLNB and radiological examinations will continue to occur and possibly even increase with improving accuracy of these techniques, their unambiguous and accurate classification will remain important. Therefore, more research on pN1b and pN2b is advised and revision of TNM classification is desirable as solitary IMLNMs may be regarded as a single category.
